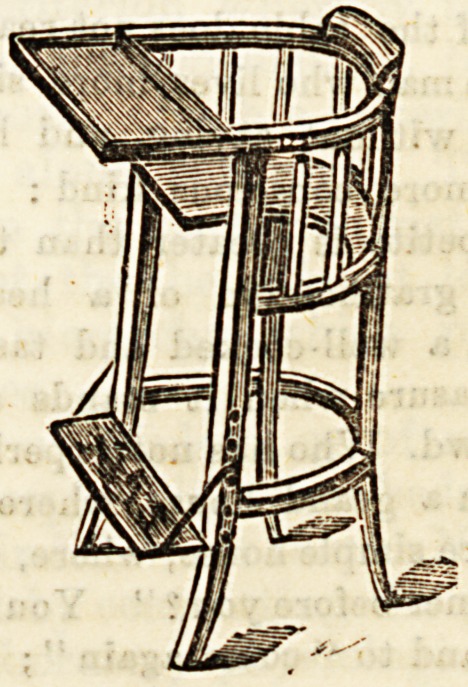# New Drugs, Appliances, and Things Medical

**Published:** 1892-03-12

**Authors:** 


### NEW DRUGS, APPLIANCES, AND THINGS MEDICAL.

[All preparations' appliances, novelties, etc., of which a notice is desired, should be sent for The Editor, to care of The Manager, 140, Strand, London, W.C.]

### NEWLY INVENTED HIP DISEASE CHAIR.

ALFRED CARTER, HOLBORN VIADUCT. (Patented 1892.)

Mr. Carter has forwarded to us a description of the above, and we give a reduced drawing of it. The object of the patentee is to enable patients suffering from hip disease to sit up in an ordinary position without disturbing the splint, and without detriment to the limb. Many patients with this disease are condemned to lie flat for years, with very little or no change of position. The inventor is hopeful that the chair will afford some relief from the monotony and tedium of remaining in one position for such a prolonged period. As will be seen from the drawing, the seat is cut out on the one side so as to admit of the splint passing inside. When the patient is to be placed in the chair, it is only necessary to remove the table and unhook the foot-rest; as soon as he is comfortably seated, the table should be replaced, and the foot-board hooked on at a suitable height for the foot of the sound limb to rest on. The patient can then amuse himself by reading, the study and practice of music, drawing, or, in fact, any occupation which can be followed in a sedentary position. The back of the chair being round, affords good support for the patient's back and sides. With these advantages we can only hope that many unfortunate subjects of hip-joint disease will be made happier by its use.

## Figures and Tables

**Figure f1:**